# Contactless photoplethysmography for assessment of small fiber neuropathy

**DOI:** 10.3389/fphys.2023.1180288

**Published:** 2023-08-23

**Authors:** Zbignevs Marcinkevics, Uldis Rubins, Alise Aglinska, Inara Logina, Dmitrijs Glazunovs, Andris Grabovskis

**Affiliations:** ^1^ Department of Human and Animal Physiology, Faculty of Biology, University of Latvia, Riga, Latvia; ^2^ Biophotonics Laboratory, Institute of Atomic Physics and Spectroscopy, University of Latvia, Riga, Latvia; ^3^ Department of Neurology and Neurosurgery, Riga Stradins University, Riga, Latvia

**Keywords:** remote photoplethysmography, imaging photoplethysmography, small fiber neuropathy, vasomotor responses, axon reflex flare, cutaneous vasomotion, optical diagnostic imaging, topical heating

## Abstract

Chronic pain is a prevalent condition affecting approximately one-fifth of the global population, with significant impacts on quality of life and work productivity. Small fiber neuropathies are a common cause of chronic pain, and current diagnostic methods rely on subjective self-assessment or invasive skin biopsies, highlighting the need for objective noninvasive assessment methods. The study aims to develop a modular prototype of a contactless photoplethysmography system with three spectral bands (420, 540, and 800 nm) and evaluate its potential for assessing peripheral neuropathy patients via a skin topical heating test and spectral analyses of cutaneous flowmotions. The foot topical skin heating test was conducted on thirty volunteers, including fifteen healthy subjects and fifteen neuropathic patients. Four cutaneous nerve fiber characterizing parameters were evaluated at different wavelengths, including vasomotor response trend, flare area, flare intensity index, and the spectral power of cutaneous flowmotions. The results show that neuropathic patients had significantly lower vasomotor response (50%), flare area (63%), flare intensity index (19%), and neurogenic component (54%) of cutaneous flowmotions compared to the control group, independent of photoplethysmography spectral band. An absolute value of perfusion was 20%–30% higher in the 420 nm band. Imaging photoplethysmography shows potential as a cost-effective alternative for objective and non-invasive assessment of neuropathic patients, but further research is needed to enhance photoplethysmography signal quality and establish diagnostic criteria.

## 1 Introduction

Pain is an evolutionarily adaptive trait that substantially improves the survival of a species, as it is a product of the nociceptive system that is closely interconnected with reward and motivation mechanisms. This results in the avoidance of potentially dangerous stimuli or activities (Walters and De C Williams, 2019). However, sometimes due to pathological conditions, pain sensations can become spontaneous and chronic, which can substantially impair the functioning and wellbeing of an individual. One particularly debilitating form of chronic pain is neuropathic pain, which refers to a specific chronic pain syndrome characterized by pain and sensory abnormalities in body parts that have lost their normal peripheral innervation or sensory representation (Costigan et al., 2009). The prevalence of the syndrome is approximately 2.4% globally, and the percentage rises with age, with 5%–7% in those aged 45 and older (Callaghan et al., 2015). This is not entirely clear and can vary in different age populations. Approximately 40% of sufferers never receive appropriate diagnosis, while 21% receive no pain management at all (Carnago et al., 2021). Usually, neuropathic pain occurs as a result of damage to small fibers (A-delta and C nerve fibers) and can be caused by a wide range of disorders (Colloca et al., 2017). Recently, a substantial worsening of the global situation has been observed due to the SarsCov-2 pandemic, which caused a COVID-19 disease burden worldwide (Pires et al., 2022). A growing number of studies have documented a wide variety of neurological manifestations associated with COVID-19 disease, particularly neuropathies, which can account for as much as 36.4% of COVID-19 patients (Ftiha et al., 2020).

The present diagnostics of peripheral neuropathies are primarily based on subjective self-assessment tests or biopsies (Scott et al., 2003). Their results partly depend on the patient’s interpretation and feedback to physicians, and therefore may be doubtful for the elderly, who are the main patient group. One such test is Quantitative sensory testing, a measure of perception in response to mechanical, thermal, and painful stimuli of controlled intensity. A more objective alternative is the invasive skin biopsy technique with subsequent histological nerve fiber density determination. However, it is uncomfortable for the patients and therefore not widely used. Nevertheless, after years of research, there is still no affordable non-invasive clinical diagnostic technique for small fiber neuropathy.

In light of the present situation in healthcare and high economic demands (Breivik et al., 2013), the development of alternative techniques for objective and non-invasive diagnostics of neuropathy is of great importance. Recent studies shed light on this issue, suggesting that derangement of small nerve fibers has local manifestation on the adjacent skin and its vasculature (Ando et al., 2021). The skin is the largest human organ, which is extensively vascularized and innervated, manifesting different pathological conditions of local and systemic origin (Leal et al., 2021), such as septic shock, diabetes, hepatitis and rheumatoid arthritis. The skin is easily accessible for optical techniques and has desirable and well-known optical properties (Bashkatov et al., 2011), hence the heterogeneous non-uniform structure possessing sophisticated and not entirely understood regulatory mechanisms (Slominski et al., 2015).

Studies suggest that alterations in dermal blood flow (cutaneous vasomotor responses) evoked by different provocation tests, such as topical skin heating (Minson et al., 2011), cooling, reperfusion during post-reactive hyperemia, or iontophoresis of vasoactive substances into the skin, are promising diagnostic indicators (Lenasi, 2011). Another encouraging but methodologically challenging avenue would be spectral analyses of spontaneous oscillations of cutaneous perfusion, referred to as cutaneous flowmotions (Rossi et al., 2005), which can reflect different local and systemic regulatory mechanisms and might reveal pathology at early stage (Sun et al., 2013). All aforementioned measurements require a simple and reliable, artifact-proof technique for cutaneous perfusion monitoring. Nevertheless, most of the preceding studies on cutaneous blood perfusion were performed by laser Doppler imaging technique (Harrison et al., 1993; Cracowski and Roustit, 2016), which is sophisticated and expensive with relatively low temporal resolution (Merla et al., 2008; Rajan et al., 2009). In recent years, the contactless modality of photoplethysmography has gained popularity as a simpler and cost-effective alternative to Doppler imaging (Hagblad et al., 2010; Mizeva et al., 2015; Rodrigues et al., 2019). It has proven its capability in different diagnostic scenarios, such as monitoring local anesthesia (Rubins et al., 2010), assessing oral mucosa health (Rubins et al., 2019), diagnosing gingivitis (Marcinkevics et al., 2020), and assessing cutaneous vasomotor responses (Trumpp et al., 2016; Marcinkevics et al., 2019). The approach is similar to the conventional contact manner reflection-type photoplethysmography (Hertzman, 1937; Allen, 2007), with the photodetector being replaced by a video camera (Huelsbusch and Blazek, 2002). This allows remote registration of a large area of interest at relatively high spatial and temporal resolution (Cheng et al., 2022) while avoiding any pressure or attachment on the skin, which can prevent tissue compression, capillary blood flow occlusion (Sun and Thakor, 2016), and discomfort during the measurement (Desquins et al., 2022). By using advanced signal processing algorithms, the blood perfusion-related signal can be extracted from the subtle pixel intensity changes in the image sequence even during non-stationary position of body (Maity et al., 2022). The major technical advantages of the contactless plethysmography approach are its flexibility and scalability, which permit its extension to multispectral modality. Over the past decade, several studies have investigated the potential of multispectral modality in different experimental settings (Asare et al., 2011; Trumpp et al., 2016; Chen et al., 2020), including our previous research on the value of multispectral photoplethysmography for the clinical assessment of cutaneous microcirculation at two different depths (Marcinkevics et al., 2016).

Considering the recent evidence and achievements in the field of remote photoplethysmography (Sun and Thakor, 2016; Ryals et al., 2023), and the pressing need for improved healthcare technology in Post-coronavirus pandemic era (Gautam et al., 2020; Jazieh and Kozlakidis, 2020), the present study aims to develop a modular prototype of a contactless photoplethysmography system with three spectral bands and evaluate its potential for assessing peripheral neuropathy via a skin topical heating test. Two hypotheses were formulated. First, we hypothesize that neuropathic patients will exhibit significantly lower perfusion index values during the topical heating-induced vasomotor response than healthy volunteers. Second, we predict that utilizing three spectral bands will yield more valuable diagnostic information for neuropathic patients compared to using a single spectral band.

## 2 Methods

### 2.1 Design of contactless photoplethysmography system

**FIGURE 1:**
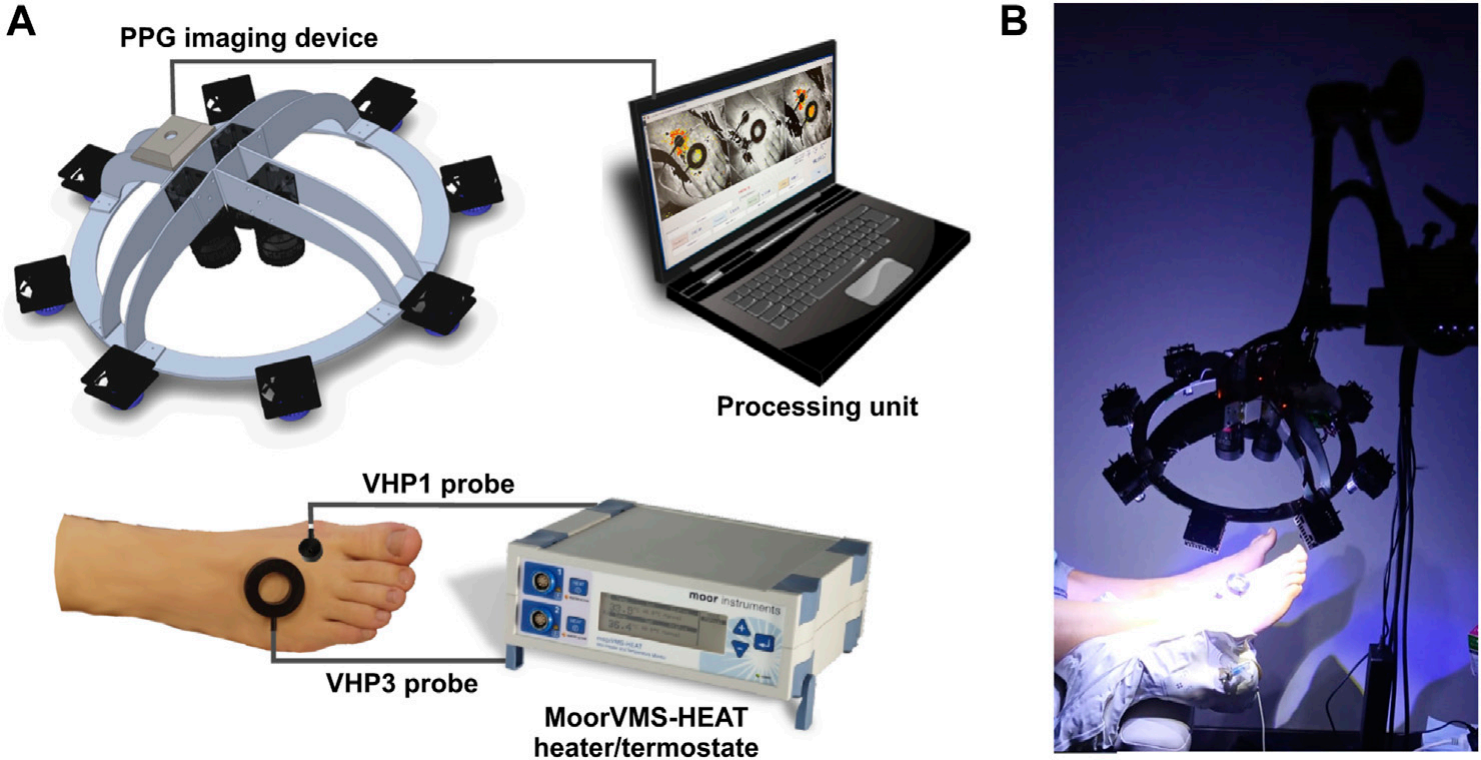
The iPPG system for cutaneous perfusion monitoring during the heating tests. **(A)** The experiment setup and major components of the system; **(B)** Photo of imaging system in action.

**FIGURE 2:**
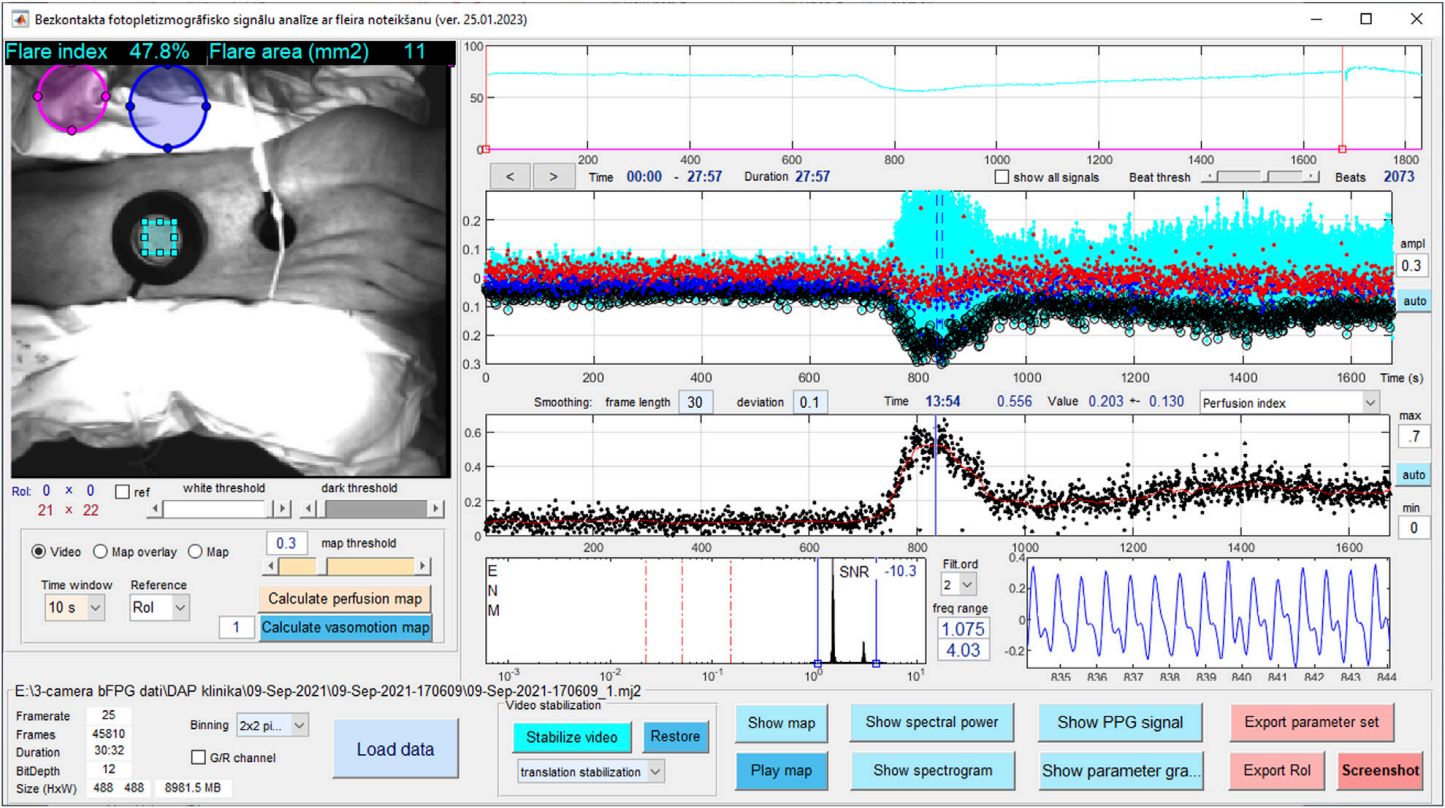
The screenshot of custom made dedicated Matlab based software for off-line data analyses.

The contactless reflection-type photoplethysmography (PPG) system prototype was designed based on our experience in developing imaging systems, current research, and clinical expert input. The system offers modularity and spectral band flexibility, allowing for customization of spectral bands to optimize measurement conditions for different tissue depths and types of biological tissue. This flexibility makes the system highly adaptable and versatile, suitable for various laboratory and clinical measurement scenarios, including cutaneous perfusion mapping and PPG waveform and phase analysis. The contactless reflection-type photoplethysmography system prototype (Figure 1) consisted primarily of three key components: an imaging device, a signal processing unit, and dedicated software for offline data analysis (Figure 2).

#### 2.1.1 The imaging device

Comprised three identical cameras (Ximea-xiQ USB-3, ADC 8–12-bits, resolution 648 × 488 pix.) equipped with the lens (Edmund Optics, C-mount f = 25 mm, F1.4, two visible spectra, and one near-infrared lens) which were mounted on the rigid aluminum frame-device chassis at fixed angles to align the visual fields of all three cameras along a single optical axis (Figure 1A). Spectral band changes were achieved by using different sets of optical narrowband interference filters mounted in front of the camera lens, along with an orthogonal polarization system. This system used one linear polarizer in front of the lens and another in front of the light source to prevent reflection from the skin surface and improve image quality. In our measurement setup, the imaging device was equipped with three narrowband filters (CW = 420, 540, and 800 nm, FWHM = 10 nm) based on pilot studies that confirmed the effectiveness of narrowband filters in improving system sensitivity by tuning reflected wavelength to particular hemoglobin absorption maxima, thus reducing biological noise in the signal. The light source consisted of eight custom-made, replaceable illuminator modules of high power LEDs, distributed in a circular arrangement on the device chassis, providing uniform illumination of the measured surface, with the option to replace and customize the desirable illumination wavelength. In the present setup, each module consisted of two blue LEDs (CW = 420 nm, FWHM = 20 nm, max. Electric power 1 W); one green LED (CW = 568 nm, FWHM = 100 nm, max. Power 1 W); and one infrared LED (CW = 810 nm, FWHM = 20 nm, max. Power 0.6 W), which were manufactured by LuxeonZ from LumiLeds (San Jose, CA, United States). Measured irradiance in the skin plane (25 cm from the illuminator) for blue at 420 nm was 1.5 mW/cm^2^, for green at 540 nm was 0.3 mW/cm^2^ and for infrared at 800 nm was 0.7 mW/cm^2^. Advanced active cooling and ultra-stabile LED driver circuit were used to maintain stable irradiation over long measurement sessions, incorporating miniature brushless fans into the back of the illuminator module heatsink plate. universal mounting options are provided by fixing the imaging system frame to the Variable Friction Magic Arm (Manfrotto), which can be attached to the optional tripod stand or any part of the bed or office table if necessary.

#### 2.1.2 The signal processing unit

To ensure high-quality video input and control of illumination modules, the imaging device was connected to a laptop computer (Intel Core i7; 16 GB of RAM) via four USB 3.0 cables. The cables were organized and protected by being enclosed in a flexible spiral tube, which helped to prevent tangling and physical damage during use. The imaging system was operated by custom-developed MATLAB-based software, which allowed for the control of cameras and video storage to 12-bit video files. Following the start of the system, the software operated in preview mode, displaying the 420, 540, and 800 nm spectral videos. When the measurement was started (by pressing the Start button), the software was switched to video recording mode, enabling the viewing of skin blood perfusion maps in each of the images. The software recorded video at 25 frames per second with a resolution of 480 × 480 pixels in 12-bit mode to achieve high dynamic range videos. At the end of the measurement, the software automatically switched back to preview mode and was ready for the next measurement.

#### 2.1.3 The data offline analyses software

**FIGURE 3:**
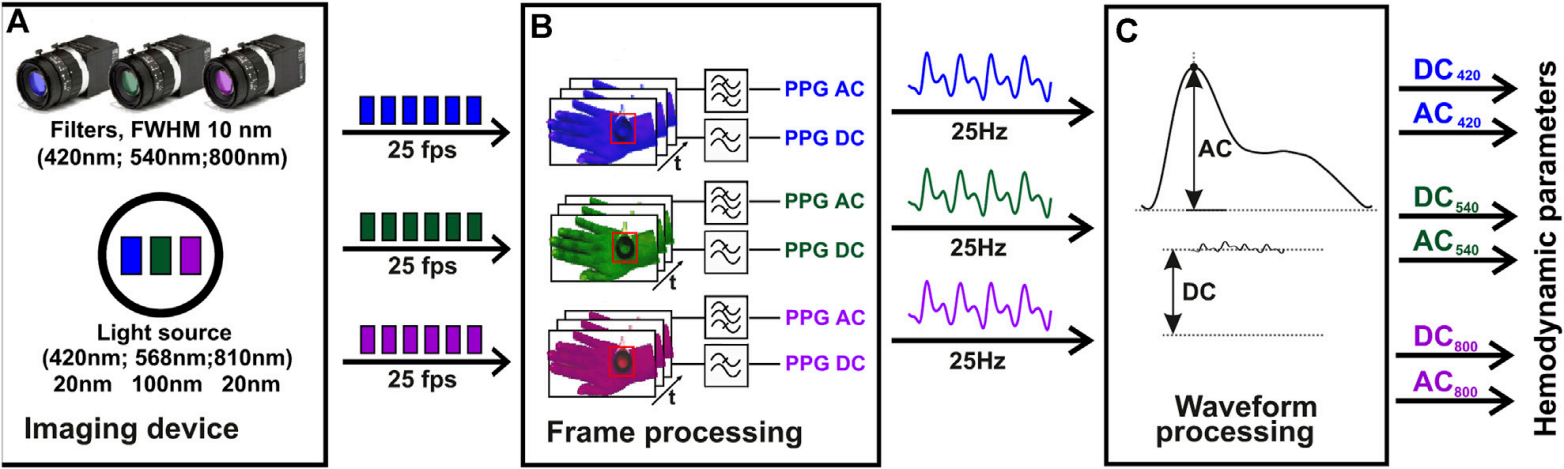
The algorithm block chart of iPPG signal analysis. The initial video data acquisition stage, employs a multi-spectral imaging device, including a multi-spectral illuminator and three cameras, each equipped with 420 nm, 540 nm, and 800 nm filters **(A)**. The second stage of the process, where the PPG signal is computed using temporal filtering of the video frames. The frequency range used for AC related to the heartbeat is (0.7–5 Hz), and for slow DC it is (0–0.3 Hz) **(B)**. The final stage, where the hemodynamic parameters, such as the perfusion index, are derived for each spectral channel using the AC and DC values calculated at each heartbeat **(C)**. Z. Marcinkevics, U. Rubins, A. Caica, and A. Grabovskis “Evaluation of nitroglycerin effect on remote photoplethysmogram waveform acquired at green and near infra-red illumination”, Proc. SPIE 10592, Biophotonics—Riga 2017, 105920E (7 December 2017); https://doi.org/10.1117/12.2297385.

The blockchart of iPPG analysis algorithm is shown in Figure 3. The most sophisticated and computationally extensive part of photoplethysmography system is Matlab platform based offline data analyses software, which contains very extensive set of contactless PPG analyses functions, such as signal filtering and frame stabilization, beat detection, estimation of characterizing waveform features, computation of augmentation, reflection and stiffness indexes, perfusion index perfusion mapping, calculation of signal Fourier spectra characteristics and analyses of vasomotor responses. The operation of software was provided by the same signal processing unit (data acquisition Laptop computer). The advantage of software is ability to operate large datasets (Big data) (∼25 GB for each measurement set) which is crucial for long, higher framerate recordings. The software is organized with interactive user interface (Figure 2). The further description of the software’s signal processing and data analysis functions which are related to vasomotor response, flare and flowmotion analyses is provided in the Signal Processing and Analyses of Cutaneous Perfusion Data sections.

### 2.2 Subjects

Thirty subjects were enrolled in the study, fifteen neuropathic patients and fifteen similar age (59.33 years vs. 57.20 years, *p* > 0.05), gender proportion (f:67%, m:33% vs. f:67%, m:33%) and body mass index (21.76 kg/m^2^ vs. 21.41 kg/m^2^) healthy volunteers. The study procedures were approved by both the Ethics Committee of the University of Latvia, Institute of Cardiology and Regenerative Medicine and Riga Stradins University Research Ethics Committee (Prot.Nr: 03.05.2018), and were in accordance with the Declaration of Helsinki (World Medical Association, 2013). Prior to the study, all subjects were informed about the protocol and gave their written informed consent.

An experienced neurologist selected our patient cohort from outpatients based on established clinical guidelines that incorporated the results of both quantitative sensory testing and neurography (sural nerve conduction test.) We applied the referent values for quantitative sensory testing thermal thresholds as proposed by Magerl et al. (2010). According to these guidelines, all patients in our cohort were diagnosed with either probable or definite small fiber neuropathy. The clinical guidelines define probable small fiber neuropathy as the presence of length-dependent symptoms and/or signs of small fiber damage in conjunction with a normal sural nerve conduction test, while the definitive diagnosis of small fiber neuropathy requires the aforementioned criteria to be accompanied by abnormal thermal thresholds detected during quantitative sensory testing at the foot and/or reduced intraepidermal nerve fiber density at the ankle, as ascertained by biopsy (Tesfaye et al., 2010; Terkelsen et al., 2017).

The control group comprised individuals who did not exhibit any length-dependent symptoms or signs of small fiber damage, and were therefore deemed to be healthy.

### 2.3 Measurement procedure

**FIGURE 4:**
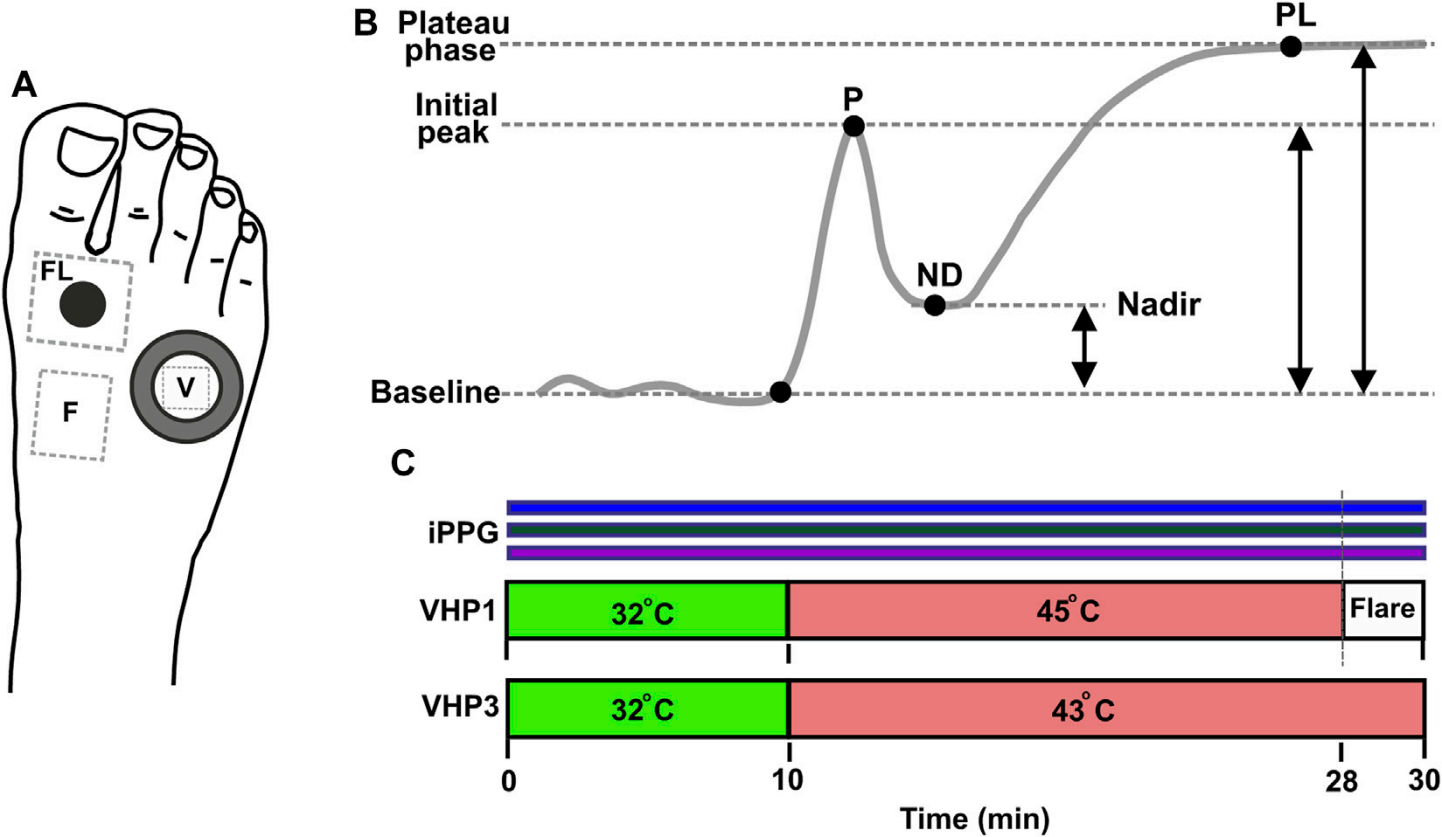
Measurement protocol. **(A)** Position of sensors and measurement regions on the dorsal aspect of foot. Water filled VHP3 heating probe is marked with large hollow ring, and the region of signal acquisition for vasomotor response trend is denoted by V; VHP1 heating probe is marked with black filled circle, flare area measurement site is denoted by FL. Flow motion acquisition region is denoted by F. **(B)** Typical trend of topical skin heating induced vasomotor response, comprising baseline, initial peak-P, nadir-ND and plateau phase–PL. Reproduced from Marcinkevics et al. (2021), licensed under CC BY 4.0 **(C)** Topical heating protocol, Simultaneous PPG recording at 420, 540 and 800 nm is marked by horizontal color bar.

To evaluate the function of small cutaneous sensory nerve fibers, we employed two modalities of a topical heating test, along with the assessment of cutaneous flow motions. The first modality aimed to produce a heating-induced vasomotor response trend characterized by biphasic changes in cutaneous blood perfusion (Kellogg, 2006). This response consists of a sharp rise (first peak) followed by a decline to a nadir, and then a subsequent increase that remains relatively constant over a longer time course, referred to as the plateau phase as shown in Figure 4B. The second modality was intended to evoke a skin topical heating-induced flare (Weidner et al., 2003), which is an extent of cutaneous reddening beyond the direct contact heating zone. Cutaneous flow motions (Kastrup et al., 1989) were acquired from intact skin regions not influenced by heating, and comprises three major spectral components (myogenic, neurogenic, and endothelial). Before the procedure, the subject was seated in a reclined position on a comfortable cosmetology seat, with adjustable spinal and leg support angles. The hands were placed on chair arm supports, and the right leg was extended and firmly fixed by a vacuum pillow to eliminate possible movements during the measurement procedure. It was ensured that all subjects were in the same position, so that the foot was approximately 10 cm below heart level. A 10-min adaptation period was allowed for subjects to become accustomed to the laboratory room conditions and the presence of the research staff. The dorsal aspect of the foot was gently wiped with an alcohol pad to remove sweat sediments and the fat layer from the skin. Two different types of heating probes were situated on the skin in the following manner (Figure 4A): the large VHP3 probe was attached to the skin using self-adhesive ring-shaped tape and filled with distilled water, so that perfusion signal can be continuously captured through water from the center of the probe. The small VHP1 probe served for inducing flare response and was gently placed on the skin, securing it with a thin rubber belt, as seen in Figure 1B. The flowmotion acquisition area was selected to avoid different influencing factors of heating-induced responses, or underlying veins and large arteries that might pulsate (Figure 4A). After placement of the probes and all adjustments to the imaging device, which is part of the contactless PPG system, were made, it was fixed to the stand and positioned approximately 25 cm from the skin so that the entire dorsal aspect of the palm fits the visual field, and the illumination is uniform in the regions of interest, providing a sharp, high-quality image.

#### 2.3.1 Heating protocol

The measurement lasted for a total of 30 min. Once the placement of VHP1 and VHP3 heating probes to the skin was complete and the measurement equipment was set up, the recording was started by pressing the record button on the custom-made dedicated software. The video capturing took place at 25 frames per second. During the data acquisition, the external illumination (regular room illumination) was switched off to avoid interference with the imaging device light source. To establish baseline perfusion at 32°C for both probes (VHP1 and VHP3), a 10-min pre-heating period was initiated at the beginning of the protocol. At the 10th minute, the probes were adjusted to 43°C for VHP3 and 45°C for VHP1 respectively as depicted in Figure 4C. The protocol then continued for an additional 18 min with the probes at their respective temperatures. After 28 min, the VHP1 probe was removed to expose the cutaneous flare area. The recording ended 2 min later, and the remaining VHP3 probe was then gently removed from the skin. To provide a visual representation of the protocol, a scheme is provided in Figure 4.

### 2.4 Signal processing

Data analysis and signal processing of video was performed offline using a dedicated Matlab software (see Figure 2). The process involved opening a previously stored measurement file and performing video pre-processing and iPPG processing. Pre-processing included three steps: 1) loading data into video buffer and spatial downsampling by factor two; 2) alignment of spectral images by estimation of geometric transformation using the “imregtform” function, and applying geometric transformation using the “imwarp” function; 3) video motion stabilization. The stabilized videos can be further analyzed to obtain haemodynamic parameters such as heart rate and perfusion index, or other parameters.

The amplitude of back-scattered light intensity pulsations fast varying component (AC) induced by heart activity is typically very small, usually below 1% from slow varying component (DC) level. Additionally, there is some fraction of biological noise present in the signal which can influence the signal-to-noise ratio and quality of signal. To address these issues, a second-order zero-phase Butterworth bandpass filter was applied within the heartbeat frequency range (0.7–5 Hz) to compute the AC signal, while the DC signal was calculated by low-pass cut-off filtering (0–0.3 Hz). The frequency ranges can be adjusted manually. To obtain hemodynamic parameters, the iPPG AC signal processing was accomplished in several steps. First, the local minima and maxima positions were found in a single PPG waveform using the built-in Matlab “findpeaks” function. If necessary, the sensitivity of the function for localization of extremal points can be adjusted manually. Then, single-period iPPG waveforms were extracted in a beat-per-beat manner, and hemodynamic parameters including pulse rate, DC signal, and AC signal amplitudes were calculated in every beat. The pulse rate was calculated using the formula:

$${Pulse\;rate}_{i}=60∙\left(t_{n+1}-t_{n}\right)/fs$$ (1)

where n is the number of the current heartbeat, t_n_ is the time of the first local minima of the pulse wave, and f_s_ is the sampling frequency of the video. Microcirculation is related to the Perfusion Index (PI), which is calculated from the AC amplitude relative to DC level in every heartbeat using the formula:

$${PI}_{n}\left(\%\right)=100∙\left({ACmax}_{n}-{ACmin}_{n}\right)/{DC}_{n}$$ (2)

where ACmin and ACmax are the minimum and maximum peak values of the pulsatile component, DC is slow-varying signal, at *n*th beat.

The strength of camera-based contactless plethysmography lies in its high spatial resolution, which is particularly useful for determining topical heating-induced flare-a region of the skin with a substantially increased perfusion above the baseline. The process involves two steps. First, a perfusion map is generated by calculating the pulsatile component in every pixel of the video using the locking-amplification principle as described by Amelard et al. (2017) as the Pearson’s linear correlation coefficient between the signal obtained in each image pixel and the ground-truth reference signal in such a way:

$$P\left(x,t\right)=\frac{\sum_{t=0}^{T}Y\left(x,t\right)R\left(t\right)}{\sqrt{\sum_{t=0}^{T}{Y\left(x,t\right)}^{2}}\sqrt{\sum_{t=0}^{T}{R\left(t\right)}^{2}}}$$ (3)

where P is a perfusion map, x is a pixel coordinate, t is a time, T is a time buffer (10 s) of a pulsatile PPG AC signal Y, and R is the reference signal which is calculated as spatially averaged Y signal from manually selected RoI. Equation 3 represents a spatially-distributed skin blood perfusion, which varies in time. Perfusion map is auto normalized, and its value varies from 0 to 1.

The area of the flare depends on the threshold, which depends on biological zero signal in the skin non-affected by external heat stimuli. The flare area is defined as the sum of the perfusion map pixels where *p* > 0.5, which we assumed as an optimum threshold considering baseline perfusion level. The total flare area (FA) is calculated as the full area minus the area of the VHP1 heater contact surface, which is 100 mm^2^. The equation for calculating FA is as follows:

$$FA=a\left(\sum_{x\in S}x-\sum_{x\in H}x\right)$$ (4)

where x is a pixel coordinate, a is the area of a single pixel, S refers to the manually selected ellipse region of interest (RoI) that covers the area surrounding the VHP1 probe, and H is the VHP1 probe contact area. The flare intensity index, which has been introduced to characterize the density of the flare, is defined as follows:

$$FD\left(\%\right)=\frac{100\cdot a}{FA}\left(\sum_{x\in S}P\left(x\right)-\sum_{x\in H}P\left(x\right)\right)$$ (5)

where P(x) is a perfusion map values in x pixels belonging to the S and H regions, FD is a flare intensity index.

#### 2.4.1 Analyses of cutaneous perfusion data

**FIGURE 5:**
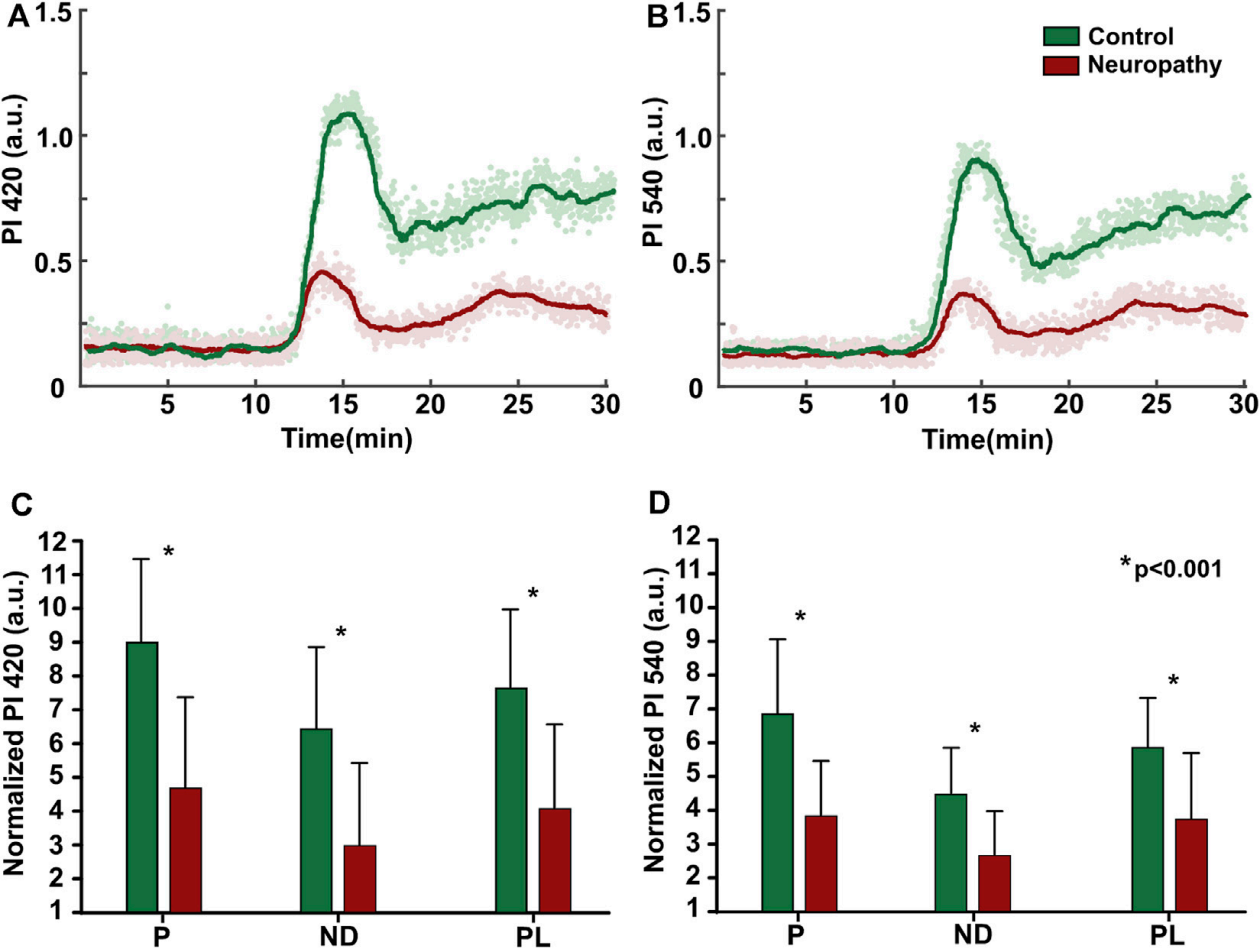
Topical heating vasomotor response trend **(A, B)** Individual example from patient and healthy subject at 420 and 540 nm illumination. **(C, D)** Baseline normalized group (patients: n = 15, control: n = 15) mean data of vasomotor response characterizing parameters; P- initial peak, ND-nadir, PL-plateau phase, values presented as mean ± standard deviation, statistically significant difference denoted by asterisk.

The characterization of heating-induced vasomotor response, heating-induced flare, and flowmotions was performed offline using data analysis software that is an integral part of the present contactless photoplethysmography (PPG) system. The relevant regions of interest were manually selected on the preview video screen, and the analyses were performed automatically. Vasomotor response was computed from the region inside the transparent part of the VHP3 heating probe, and the perfusion index changes over time were analyzed using the vasomotor response analysis module which was part of the software. The analyses incorporated perfusion index filtering and trending with the detection of characteristic inflection points that determined the amplitudes of the first peak (P), nadir (ND), and plateau phase (PL), as depicted in Figure 4B. To explore the flare response, two regions of interest were selected. First, a small circular region was selected on the contact zone of the VHP1 probe to fit directly heated skin boundaries, while the second region was placed over the larger area surrounding the VHP1 probe. The analyses were performed on a 1-min duration video fragment just following removal of the heating probe. Offline analyses included the generation of a perfusion map and the determination of the flare area, as described in the signal processing methods section. Cutaneous flowmotions were calculated from flare and heating unaffected skin by selecting a ∼40 × 40 pixel region of a 20-min duration video fragment, as depicted in Figure 5. The flowmotions were automatically divided into three spectral ranges representing the influence of myogenic (∼0.05–0.15 Hz) (Kastrup et al., 1989), neurogenic (∼0.02–0.05 Hz) (Söderström et al., 2003), and endothelial (∼0.0095–0.02 Hz) (Kvernmo et al., 1999; Kvandal et al., 2003) activity, as suggested by other studies. The averaged spectral power density for each spectral range was computed by Fast Fourier Transform using following formula:

$$PSD=\frac{1}{f2-f1}\sum_{f1}^{f2}{\left|F\left(f\right)\right|}^{2}$$

Where PSD is a averaged power spectral density, F is a Fourier transform of RoI-averaged PPG signal, f1, f2—spectral range of corresponding flowmotion component.

### 2.5 Statistical analyses

The statistical analyses were conducted using SigmaPlot 12.0 (Systat Software Inc., San Jose, CA, United States). As the majority of the data did not conform to Gaussian distribution, non-parametric statistical tests were employed. To compare the patient group with the control group, the Mann-Whitney Rank Sum Test was utilized. The baseline cutaneous perfusion values for the different wavelengths in both the control and patient groups were compared using Kruskal–Wallis One Way Analysis of Variance on Ranks (ANOVA). To reveal relationship between flare area and flare intensity index Spearman’s correlation analyses was utilized. A statistically significant difference was defined as *p* < 0.05. Unless stated otherwise, the values presented in the text and graphs are expressed as the arithmetic mean ± standard deviation.

## 3 Results

Comparing baseline amplitude of PPG waveform (AC signal) at all three wavelengths, it has been noticed that the infrared channel (800 nm) signal amplitude across all subjects did not significantly differ from the noise. Therefore, these data has been excluded from subsequent analyses, and further results are provided on blue (420 nm) and green channels (540 nm). All fifty subjects (patient group and healthy control group) displayed a reasonable amplitude PPG waveform at both channels during baseline and vasomotor response. The baseline perfusion index amplitude slightly varied among the individuals, with no statistically significant difference observed between subject groups or two PPG channels (420 nm vs. 540 nm). The mean values were as follows: 420 nm - control (0.12 ± 0.04) vs. patients (0.11 ± 0.06) and 540 nm - control (0.13 ± 0.06) vs. patients (0.14 ± 0.07).

The quantification of heating-induced cutaneous response was performed on the two separate modalities as an amplitudes of vasomotor trend and extend of cutaneous reddening area-flare.

### 3.1 Topical heating vasomotor response trend

Control group subjects exhibited a similar trend of vasomotor response at both 420 and 540 nm, with its characteristic shape comprising sharp, increase of perfusion (P1) with the following decline, nadir, and succeeding elevation, which remained relatively unchanged until the end of recording, known as the plateau phase, as shown in Figure 5. However the signal at 420 nm PPG band was 20%–30% larger, than that of 540 nm, regardless of subjects group. Meanwhile, the neuropathic patient group demonstrated a blunted response (50%), with altered initial peak (P1), nadir (ND), and plateau phase (PL), regardless of the illumination wavelength, Figure 5.

### 3.2 Topical heating flare response

**FIGURE 6:**
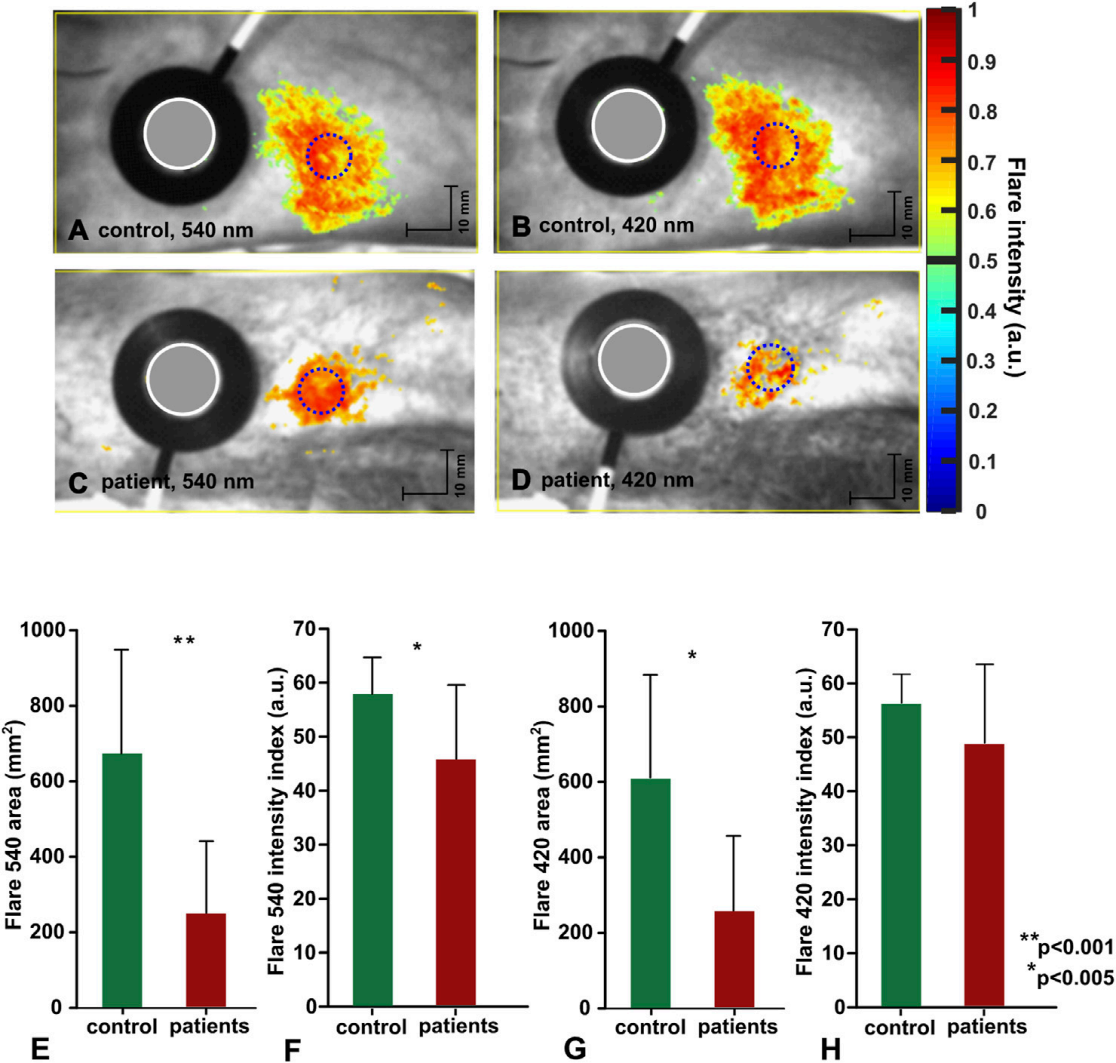
Topical heating induced flare response. **(A–D)** representative data from individual subject, flare discrimination threshold is set to 0.5. **(E–H)** Group mean data (control:n = 15, patients:n = 15) mean ± std, statistical significance denoted with asterisks.

Topical heating induced the reddening (flare) surrounding the direct contact site of the heating probe in all subjects, with some individual differences in the intensity of flare (flare intensity index) and area noticed among the same group subjects (Figure 6). Similar to vasomotor response amplitude, flare area (63%) and flare intensity index (19%) were significantly decreased in patients compared to healthy volunteers, regardless of the illumination wavelength. The patient group (n = 15) flare intensity index and flare area at 420 nm and 540 nm were as follows: 420 nm illumination 48.74 ± 14.56 a.u. and 248.46 cm^2^ ± 198.08 cm^2^; 540 nm illumination 45.89 ± 13.69 a.u. and 246.13 cm^2^ ± 195.18 cm^2^. The control group flare intensity index and flare area were: 420 nm illumination 56.26 ± 5.31 a.u. and 598.07 cm^2^ ± 271.89 cm^2^; 540 nm illumination 57.92 ± 6.77 a.u. and 673.60 cm^2^ ± 275.16 cm^2^.

### 3.3 Cutaneous flowmotions

**FIGURE 7:**
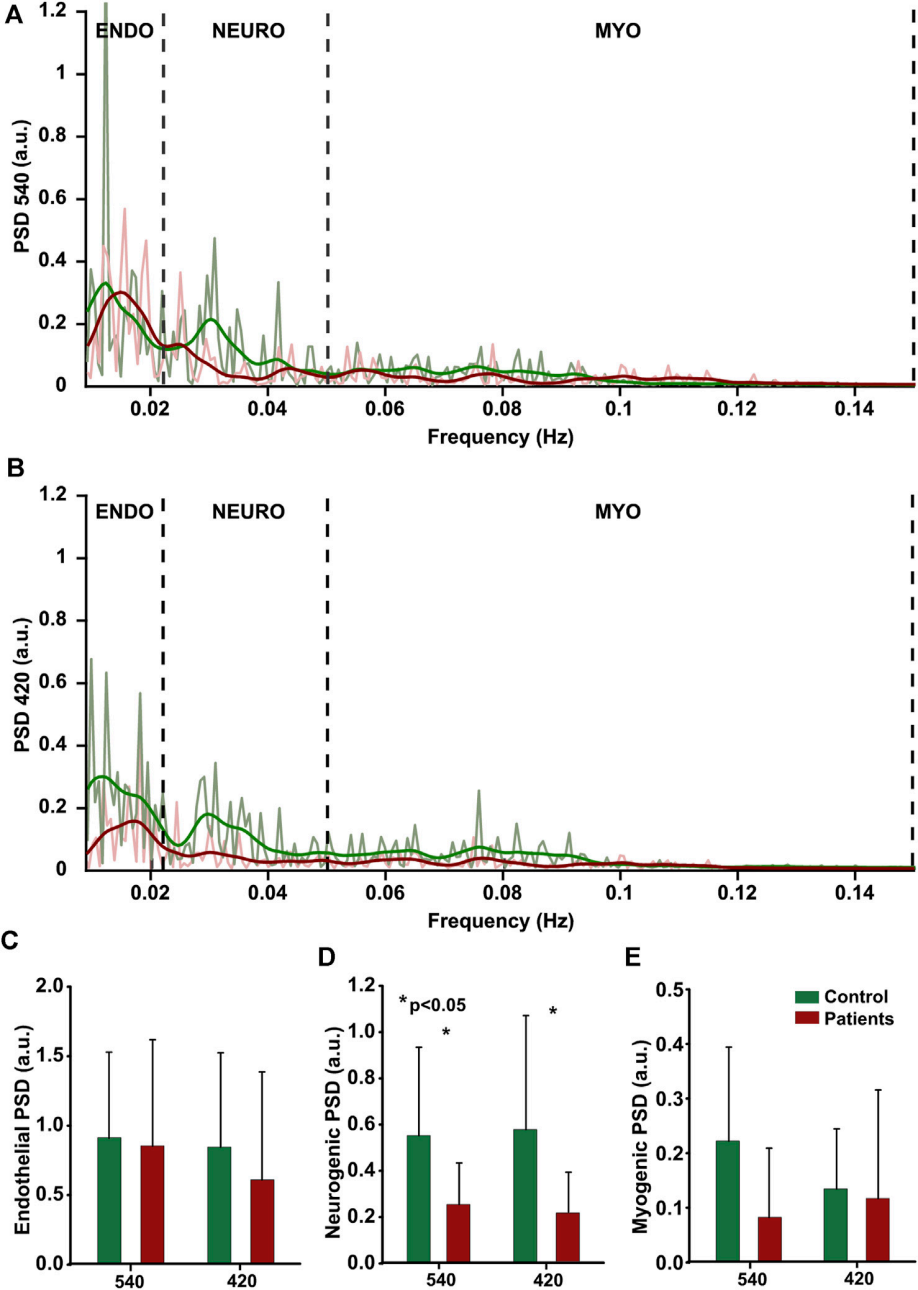
Cutaneous flowmotions. Power Spectral Density of endothelial (0.009–0.022 Hz), neurogenic (0.22–0.05 Hz) and myogenic (0.05–0.15 Hz) components at two wavelength, **(A, B)** individual data from one subject. **(C–E)** group mean data (control:n = 15, patients:n = 15), statistically significant data denoted by asterisks.

Acquisition of cutaneous flowmotions was more challenging due to the requirement of long duration artefact-free recording; therefore, moderate substantial variation among the same group subjects was observed for all three components, regardless of illumination wavelength. A noteworthy finding is the significantly larger (54%) neurogenic component of the control group compared to the neuropathic patient group at both wavelengths (Figure 7). The group mean values for healthy volunteers were as follows: at 420 nm illumination-endothelial 0.85 ± 0.68 a.u., neurogenic 0.58 ± 0.49 a. u., myogenic 0.14 ± 0.11a.u.; at 540 nm illumination-endothelial 0.91 ± 0.62a.u., neurogenic 0.55 ± 0.38 a. u., myogenic 0.11 ± 0.08 a. u. Group mean values for neuropathic patients were as follow: at 420 nm illumination-endothelial 0.85 ± 0.68 a. u., neurogenic 0.58 ± 0.49 a. u., myogenic 0.14 ± 0.11 a. u.; at 540 nm illumination-endothelial 0.91 ± 0.62 a. u., neurogenic 0.55 ± 0.38 a. u., myogenic 0.11 ± 0.08 a. u.

## 4 Discussion

Over decades there were several attempts to use photoplethysmography for assessment on neuropathies, ranging from PPG waveform parameter analyses (Bryce et al., 2022) to spectral analyses of PPG signal fluctuations (Bentham et al., 2018), and using single point PPG recording and multi-channel recording (Kim et al., 2007), alone or in combination with different modalities such as ECG or laser Doppler (Kim et al., 2008). However most of the studies up to date in this field were performed using contact manner conventional transmission type PPG, which is usually applied in the fingers and there are sparse studies assessing function of peripheral nerve fibers by contactless modality of PPG. The present study makes a significant contribution to the evaluation of multispectral imaging photoplethysmography for assessing neuropathic patients. By examining cutaneous flow motions and topical heating-induced vasomotor responses, we demonstrate the capability of this method, which to the best of our knowledge is the first of its kind. It was expected that a multispectral approach, comprising three different wavelengths, may provide additional diagnostic information, and therefore was implemented in our present setup. Contrary to our expectations, it was possible to obtain reliable PPG signal only at two (420 and 540 nm) out of three wavelengths during the same test of topical heating, as the signal at 800 nm illumination was extremely noisy and did not significantly differ from the biological zero level. This finding is in contrast to our previous studies where a detectable, hence smaller amplitude signal was obtained from both the dorsal and palmar aspect of the hand at the baseline and during topical heating using similar wavelength illumination (Marcinkevics et al., 2016; Marcinkevics et al., 2017), albeit with approximately four times larger spectral bandwidth, which covers a broader range of chromophores providing absorption in adjacent wavelengths. The possible reasons for the diminished PPG signal at this wavelength are low Hb absorption and lower density of arterio-venous anastomoses in the cutaneous vasculature of the foot’s dorsal aspect, which counterweights our initial idea of selecting the Hb isosbestic point, which might be indifferent to alterations of Hb saturation and could provide information regardless of the patient’s arterial oxygen content. Noteworthy was finding that baseline perfusion was similar in the blue (420 nm) and green (50 nm) PPG channels and did not differ between neuropathic and control group, which allowed us to normalize cutaneous perfusion to the baseline. Different normalization approaches have been mentioned in the early studies for inter-subject comparison, including normalization to maximal vasodilatory capacity, which is achieved by pharmacological intervention, such as iontophoresis of sodium nitroprusside used or acetylcholine, contributing as an endothelium independent vasodilators (Kellogg et al., 1999), first being an NO dependent, and second acting directly on smooth muscle cells. Or physiological normalization, such as maximal reperfusion during post occlusive reactive hyperemia (Shirazi et al., 2021) and topical heating induced vasodilation (Chaseling et al., 2020). Another option could be normalization to the baseline, which is more non-intrusive but feasible only if constant initial baseline conditions are achieved. All mentioned approaches have its own limitations therefore simpler and less intrusive baseline approach was presently utilized. Its validity can be supported by the fact that all subjects regardless age and health state exhibited similar absolute values of perfusion index during the baseline, resulted by preconditioning of 32°C preheating.

### 4.1 Heating induced vasomotor response trend

All subjects responded to topical heating, producing well-known three-phase pattern of cutaneous vasodilation: an initial peak within the first 5–6 min and a subsequent nadir followed by a sustained plateau (Kellogg et al., 1999; Minson et al., 2001). Neuropathic patients showed diverse response with significantly reduced amplitude of cutaneous vasodilation in both PPG channels, particularly in the initial vasodilatory peak (approximately 58%) and to a moderate extent in the plateau phase (approximately 45%). This observation was consistent across all subjects, regardless of the PPG channel, with some individual variations. The interpretation of this phenomenon is particularly challenging, as there is a lack of studies on contactless PPG assessment of neuropathies. However, our recent study evaluating reversible pharmacological impairment of cutaneous sensory nerve fibers using remote photoplethysmography showed a decrease in the initial vasodilatory peak, but unaltered plateau phase (Marcinkevics et al., 2021), which is contrary to the present finding. Similar were provided from neuropathic patients studies using laser Doppler technique (Kilo et al., 2000; Krishnan and Rayman, 2004; Carter and Hodges, 2011; Obayashi and Ando, 2014; Kubasch et al., 2017), hence the technique substantially differs from PPG. The decreased vasodilatory response in the neuropathic group may be explained by underlying physiological mechanisms of heat induced vasodilation. The literature suggest that major contributor to transient initial vasodilatory peak is a local sensory nerve-mediated axon reflex (Minson et al., 2001), mediated by TRPV1 channel dependent activation of C-fiber afferent nerve fibers that release substance-P and calcitonin gene-related peptide (GCRP) with a modest contribution of NO during early phase (Marche et al., 2017) and therefore may reflect both endothelial and small nerve fiber function. Studies suggest slightly diminishing initial peak due to the aging (Millet et al., 2012), and equally sympathetic-parasympathetic balance along to hormonal level could influence response, potentially accounting to observed variance in our subjects groups. Nevertheless, the substantial effect of age on vasomotor response is excluded, as the patient and control groups have similar age structure, confirmed by statistics. The possible explanations to reduced plateau phase in our patient cohort, is contribution of plethora of mechanisms in its genesis. There is evidence that plateau is only 60%–70% endothelium NO-dependent (Fieger and Wong, 2010; Bruning et al., 2012; Fujii et al., 2014), and could be modulated by other factors such as adenosine receptors, endogenous reactive oxygen species (Huang et al., 2012) and transient receptor potential vanilloid type 1 (TRPV1) channels which are expressed on the membrane of sensory nerve fibers (Wong and Fieger, 2010) and could be deranged in neuropathic patients. In addition etiology of peripheral neuropathies is multifaceted, frequently related to endothelial dysfunction such as in diabetic polyneuropathy. Clinical studies have shown that endothelial function assessed by endothelium-dependent vasodilation is impaired in diabetic patients (McVeigh et al., 1992) although the pathogenesis has not been fully elucidated. Our neuropathic group was etiologically heterogeneous and impairments of endothelial function cannot be ruled out.

### 4.2 Flare response

Another more robust expression of topical heating is the flare response-a type of localized neurogenic inflammation manifested as the reddening of the skin region which surrounds directly heated site. In our study, the flare response along the vasomotor response trend was employed to evaluate cutaneous nerve fiber function in neuropathic patients. The essential finding of present photoplethysmography study is reduced flare area (approx. 63%) and flare intensity (approx. 19%) in neuropathic patients in comparison to control group regardless of the illumination wavelength. The similar effect have been showed by several laser Doppler studies utilizing different provocation procedures, such as topical skin heating and vasoactive substance iontophoresis in neuropathic patients (Krishnan and Rayman, 2004; Bickel et al., 2009; Green, 2009; Green et al., 2009; Illigens et al., 2013; Namer et al., 2013; Sharma et al., 2015; Abraham et al., 2016; Calero-Romero et al., 2018). However there are also controversial studies, suggesting decreased flare area in neuropathic patients with structurally deranged nerve fibers (Bickel et al., 2009), while others points on flare reliability only for painful neuropathy conditions (Ysihai, 2009). Notably, our present research revealed that the flare intensity index in neuropathic patients remained unchanged regardless of the size of the flare area. What is in line to the laser Doppler study by Alistair et al. suggesting correlation of flare intensity to microvascular function and correlation of flare area to small fiber functions (Arnold et al., 2015). And in the light of this study the slight decrease of flare intensity index might point on moderate microvascular impairments in our neuropathic patient group, which is consisted with findings related to diminished plateau phase of these patients. Hence, it is difficult to interpret the present data due to the lack of relevant studies and sparse evidence regarding photoplethysmography’s ability to assess neuropathic patients using quantification of flare response, as this imaging modality differs substantially from Laser Doppler technique and results cannot be directly attributed to other imaging modalities without appropriate investigation. Nonetheless, there is some evidence from our previous research (Marcinkevics et al., 2021) on potential of imaging photoplethysmography for assessment of small fiber functions in the healthy volunteers, which may be extrapolated to the neuropathic patients. Recent evidence suggest that pathophysiology of small fiber neuropathy is related to spontaneous activation of small unmyelinated sensory polymodal nerve fibers which are aimed to detect and transmit temperature and slow pain to the central nervous system; hence, when impaired, can cause chronic neuropathic pain. In the healthy individuals topical skin heating (above 42°C) depolarizes small unmyelinated dermal C-fibers, resulting in afferent action potentials that are conducted toward the spinal cord and, at branching points, antidromically invade peripheral branches adjacent to the initial stimulation point, triggering release of vasoactive substances, such as substance P and calcitonin gene-related peptide (CGRP), from nerve terminals, which leads to arteriolar smooth muscle relaxation and vasodilation at the localized skin area extending outside heated skin region (Weidner et al., 2003). While in the neuropathic patients deranged nerve fibers produce substantially blunted response, which can be detected by imaging photoplethysmography, Laser Doppler imaging, and likely any imaging technique with potential to measure cutaneous blood perfusion.

### 4.3 Flowmotions

Spectral analysis of photoplethysmography (PPG) signals has long been a desirable technique due to the relative ease of recording and the potential for advanced mathematical tools to yield valuable diagnostic information. In the recent study, we have expanded upon this approach and performed a comprehensive evaluation of the efficacy of contactless PPG in the assessment of patients with small fiber neuropathy using cutaneous flowmotion analyses. The key finding of our present study is a marked reduction in the neurogenic component of vasomotor responses in patients with neuropathy, which is consistent with the observed reductions in flare area, flare intensity, and initial peak pointing on deranged small nerve fiber function. Surprisingly, there was no statistically significant difference between the endothelial flowmotion component in healthy subjects and neuropathic patients, despite the latter group showing a reduced plateau phase in vasomotor response, which can be partly explained by contribution of systemic factors or insufficiently deranged endothelial function of patients. Overall, our results are consistent with other studies (Bernardi et al., 1997; Lefrandt et al., 2003; Meyer et al., 2003; Quattrini et al., 2007; Sun et al., 2013; Körei et al., 2015) that have used Laser Doppler to evaluate diabetic patients with neuropathy, as well as with our previous study that utilized photoplethysmography to assess small cutaneous nerve fibers in healthy subjects (Marcinkevics et al., 2021). However, the interpretation of our present findings is constrained by the lack of literature on flowmotions studies that utilize the same spectral analysis approach (three major spectral components) and photoplethysmography, as most existing research on this topic are conducted with the Laser Doppler technique, emphasizing the necessity for further research using comparable methodologies to validate and expand our results. Furthermore, the interpretation of our results is limited by incomplete knowledge of the genesis and influencing factors of flowmotions. The precise mechanism underlying these oscillations remains unclear, but prior research suggests a local origin stemming from dynamic interactions between sympathetic vasoconstriction, pressure-dependent vasoconstriction, flow-dependent endothelium-mediated vasodilation, metabolic vasodilation, and spontaneous myogenic activity (Rossi et al., 2006), which can indirectly support our recent findings on alteration on neurogenic component in neuropathic patients.

### 4.4 Study limitations

While the study was conducted with a commitment to precise methodology and strict adherence to the experimental design, certain limitations were encountered that could impact the reliability of the findings, necessitating careful interpretation of the results.

First, the generalizability of our findings could be affected by the relatively small sample size and the diverse nature of the neuropathic patients we studied. Nevertheless, significant results were still achieved, underscoring the robustness of our photoplethysmographic evaluations.

Second, our study primarily focused on functional assessments of neuropathic patients, without a direct morphologic evaluation of nerve fibers by biopsy to validate clinical diagnoses. However, the intent of our study was not to provide structural assessments, but to showcase a potential functional evaluation technique that could augment existing structural testing methods.

Third, there exists the possibility of parameter drift in the photoplethysmography imaging system during recording, which could potentially affect the recording of vasomotion due to their low frequency. To mitigate this, we assessed the stability of light sources prior to the experiment. Our data showed that for all three channels, the standard deviation did not exceed 0.05% of the mean PPG signal in the 0–5 Hz frequency range. Moreover, the power spectral density (PSD) of the PPG signal, calculated from a white reference video in the 0.0095–0.15 Hz frequency range, was two orders of magnitude lower than the PPG signals obtained from healthy subjects. This indicates that any potential instability of the LED did not significantly influence the flowmotion measurements.

Fourth, the slow respiration rate of subjects could potentially affect the myogenic component of flowmotions (Liu et al., 2020). However, we did not control for this as we assumed subjects were breathing normally in either sitting or resting positions (Rodríguez-Molinero et al., 2013; Miles-Chan et al., 2014; Katz et al., 2018). Further, normal breathing does not interfere with the myogenic frequency range or affect the PPG signal. As corroborated by other studies of vasomotion using laser Doppler, the control of respiratory rate during cutaneous blood perfusion recording is not a critical factor for measuring vasomotion components (Kastrup et al., 1989; Meyer et al., 2003).

Despite these limitations, we believe our study makes valuable contributions to the literature and opens avenues for future research.

### 4.5 Conclusive remarks

Overall, our study highlights that photoplethysmographic evaluation of the flare response is the most methodically simple and robust technique among all we tested. While the analysis of flowmotions may appear simple and attractive, but is less informative due to its sensitivity to slow fluctuation artifacts and measurement site variations. Taken together, these results suggest that photoplethysmographic evaluation of the vasomotor flare response holds promise as an objective clinically valuable tool for assessing small fiber function in neuropathic patients.

One partially unresolved issue in our study concerns the diagnostic utility of the multispectral approach in photoplethysmography. Our findings suggest that there are no clear indications for any additional diagnostic information gained from the simultaneous use of the green (540 nm) and blue (420 nm) channels, nor are there any strong implications for the preference of the blue channel over the green in assessing neuropathic patients. However, we did observe that in the blue channel, the vasomotor response was numerically larger by approximately 20%–30% in both the patient and control groups, which could be attributed to the higher hemoglobin absorption at this wavelength (Zijlstra et al., 1991) and slightly different penetration depth into the skin, comprising different density of vessels (Finlayson et al., 2022). Nonetheless, despite these differentiating factors, both 420 nm and 540 nm light penetrate only superficially at the epidermal-dermal junction where the majority of vessels are densely situated capillary loops without substantial contractile elements, the two wavelengths provide a similar pattern of vasomotor response. These results suggest that the application of the two band approach in photoplethysmography may not yield significant diagnostic value in the evaluation of neuropathic patients and the usefulness of different PPG bands in diagnostics is highly debatable, while several studies emphasize the contrary (Asare et al., 2011; Chen et al., 2020), such as the study by Labuda et al. (2022) The argument put forth by the authors is that information can be extracted from different depths. At the outset of our study, we hypothesized that simultaneous recording at different illuminations would provide valuable diagnostic information from varying vascular layers within the skin due to the wavelength-dependent penetration depth of light. However, our findings failed to support this hypothesis. An explanation for this may be found in the genesis mechanisms of photoplethysmography, which, despite decades of research and widespread application varying from heart rate monitors to intensive care pulse oximeters, have not been fully elucidated. Several recent studies have advocated the classical, well-accepted volumetric photoplethysmography model (Moço et al., 2018), which posits that the PPG signal originates from volume variations in the arteriole-arterial network at different depths. According to this model, the depth-origin of PPG using green wavelengths is dermal blood volume variations, while red-IR wavelengths may interact with subcutaneous blood volume variations. Another, the red blood cell aggregation model (Fine and Kaminsky, 2022), suggests that the source of the optical signal pulsation is associated with the modulation of the scattering of RBCs in the blood vessels that is caused by the modulation of blood flow velocity. The change in blood scattering can be explained by the change in the average size of aggregates following the fluctuations of shear forces, which vary during the course of the pulse wave. Recently, an interesting tissue compression model was proposed by Kamshilin et al. (2015), which states that pulse oscillations of the arterial transmural pressure, which occur during every cardiac cycle, deform the connective-tissue components of the dermis. Therefore, further studies using advanced tissue modeling are necessary to reveal a more unified mechanism of PPG signal origin that would fit all existing hypotheses and experimental data.

## 5 Conclusion

Overall, this study demonstrate the potential of imaging photoplethysmography as a cost-effective and straightforward alternative to existing imaging techniques for the assessment of neuropathic patients, providing novel information to the field of chronic pain diagnostics. However, in order to effectively implement this technology in clinical settings, further extensive research is required to improve provocation methodology, enhance PPG signal quality, and establish diagnostic criteria and referent values for heat-induced vasomotor tests, which can be achieved through the utilization of novel approaches, such as deep learning.

## Data Availability

The raw data supporting the conclusions of this article will be made available by the authors, without undue reservation.
